# Supplementary material for: High-performance energy harvesting and continuous output using nylon-11/BaTiO₃-PVDF triboelectric nanogenerators with strong dielectric properties
Source: PLoS One. 2025 May 20;20(5):e0319802. doi: 10.1371/journal.pone.0319802 (PMC12091776; doi:10.1371/journal.pone.0319802)
Supplement: Attachment — Submitted filename: Responses .docx [file pone.0319802.s002.docx]

Dear Editor：

Thank you very much for forwarding the reviewers’comments on our Manuscript

(Submission ID: 88f1708d-e0f4-418a-97ec-71818a932804) to us. We thank the

reviewers for their critical reviews of our manuscript. We are pleased that the

reviewers recognize the value of our work. Below please find our responses to the

editor’s and reviewers’comments.

Best regards,

Sincerely,

Dr.Nurulazlina

Faculty of Engineering, Built Environment and Information Technology

Segi University

-------------------------------------------------------------------------------------------------------

**Response to Reviewer 1**

**Comment-1**

The introduction should include the advantages and limitations of the TENGs reported in the literature. Furthermore, this section must consider the scientific contribution, advantages, or novelty of the proposed TENG.

**Response:** We feel great thanks for your professional review work on our article. According to your nice suggestions, We have corrections to our previous draft in introduction with red:

TENGs limit their use due to lower output power density, and low conversion efficiency makes it difficult to drive loads(*2024,Micromachines, 15(9), 1114*).

BaTiO₃promotes better crystallization of PVDF. in addition to its own good electrical properties, BaTiO₃ has better performance as a composite(*2024,Nano Energy, 122, 109264*).

Specifically, this paper presented the fabrication a new TENG of high-performance, highly flexible using nylon-11 and PVDF with BaTiO₃NWs.

**Comment-2**

This manuscript must consider the design phase of the proposed TENG, considering detailed descriptions (e.g., figures) of its dimensions, materials, and performance.

**Response:** We sincerely thank the editor and all reviewers for their valuable feedback that we have used to improve the quality of our manuscript. We have corrected it in the manuscript with red. Fig. R1(a) shows a schematic diagram of the device and the fabricated TENG. When the nylon-11 and the PVDF-BTONWs are contacted under pressure, a surface charge transfer occurs in the contact area due to the triboelectric effect, where the positively charged layer of the nylon-11 (top) loses electrons to carry a positive charge, while the negatively charged layer of the PVDF-BTONWs (bottom) gains electrons to carry a negative charge. After the separation of the nylon-11 and the PVDF-BTONWs, a potential difference between the top and bottom electrodes occurs due to electrostatic induction, which induces electrons to flow from the bottom electrode to the top electrode, forming a half-cycle negative current pulse. When the nylon-11 and PVDF-BTONWs are pressed, a reverse current pulse, i.e., a half-cycle positive current pulse, is generated. Repeatedly, the external circuit could generate AC during each pressing and releasing cycle, as shown in Fig. R1(b). The most TENGs structure are the vertical separation type*(Nano Energy, 2024,122, 109264)(Results in Materials,2024, 22, 100576).*

Fig. R1. (a) Schematic diagram of the device assembly structure. (b) The working mechanism of TENG.

Than, The BTONWs-PVDF nanofibers were deposited on an aluminum foil using electrostatic spinning, and the resulting sample was cut into 4 × 4 cm² squares. Subsequently, the squares were fixed to laser-cut 3 mm thick acrylic substrates by using double-sided adhesive tape to tightly fit the side with the nanofibers (the aluminum foil as the backing layer) to the acrylic plate, so that the aluminum foil served as both a mechanical support layer and an electrode. In order to induce an electrical signal, a copper wire with a diameter of about 0.51 mm was connected to the aluminum foil, and a transparent PET film with a thickness of 0.5 mm was used as a contact-separation friction interface (as shown in Fig.R2.). This designed structure not only simplifies the device assembly steps, but also lays the foundation for subsequent performance evaluation and application testing.

Fig. R2. (a)-(d) The preparation process of BTONWs-PVDF nanofibers. (e) The designed of TENG. (f) Digital picture of assembled TENG.

PVDF exhibits exceptional polarization capabilities, which contribute to enhancing the storage and transfer efficiency of triboelectric charges. It possesses a high dielectric constant, excellent flexibility, and mechanical properties, enabling it to adapt to various deformation requirements, making it well-suited for wearable devices and flexible electronics. Furthermore, PVDF demonstrates strong resistance to humidity, temperature fluctuations, and chemical corrosion, ensuring its suitability for long-term applications. Due to its strong negative triboelectric properties, PVDF has been widely used in TENG devices to effectively capture positively charged triboelectric charges, thereby improving output performance *(Advanced Materials, 2019, 31(40), 1808487)*.

Nylon-11 exhibits favorable triboelectric properties, displaying significant positive triboelectric polarity when in contact with negatively charged materials. It also possesses excellent wear resistance and toughness, enabling it to maintain stable performance under repeated friction. Nylon-11 is widely employed as the positive electrode material in TENGs due to its pronounced positive polarity, forming a significant potential difference with PVDF, which enhances the efficiency of triboelectric charge separation *(Nano Energy, 2017, 37, 1–24)*.

The electrical performance was measured using a Keithley 6514 electrometer, which is highly sensitive and particularly suitable for detecting weak signals (Advanced Energy Materials, 2017, 7(23)). The output power was calculated in real time based on the formula P=UI, where the measured voltage (U) and current (I) were used for the calculation(*Advanced Materials 2023, 35, 2208139*; *Advanced Materials Technology 2019, 1900905*; *Microelectronic Engineering 2022, 257, 111725*).

**Comment-3**

The authors must regard more detailed information on the fabrication process of the TENG.

**Response:** We think this is an excellent suggestion. Below is the detailed fabrication process of the TENG, which we have partially modified in the original draft with red.In this study, BTONWs-PVDF nanofibers were first deposited on aluminum foil using electrostatic spinning. Pour the precursor into a 10 mL syringe made with a polypropylene barrel, 21 G stainless steel needle, and PTFE tubing. Injections were made at a rate of 1 ml per hour on a syringe pump. The needle was mounted on a reciprocating moving platform and a voltage of 21 KV was applied to the needle with a distance of 17 cm from the needle to the drum collector for collection, and the drum collector was rotated at 1500 rpm. The resulting samples were cut into 4x4 cm². Subsequently, the square was fixed on a laser-cut 3 mm thick acrylic resin substrate, and the side with the nanofibers (the aluminum foil as the bottom layer) was tightly attached to the acrylic plate using double-sided adhesive, so that the aluminum foil served as both a mechanical support layer and an electrode. In order to induce an electrical signal, a copper wire with a diameter of about 0.51 mm was connected to the aluminum foil, and a transparent PET film with a thickness of 0.5 mm was used as a contact-separation friction interface (as shown in Fig. R3.). Finally, the copper wire was connected to a Keithley Model 6514 electrical test system to characterize the electrical output of the device, including parameters such as current and voltage. This designed structure not only simplifies the device assembly steps, but also lays the foundation for subsequent performance evaluation and application testing.

Fig. R3.Physical image of TENG.

**Comment-4**

The size of Figure 3(f,g,h) is small. In addition, the resolution of this Figure must be enhanced.

**Response:** According to reviewers comments, Considering that Fig. 3 consists of multiple figures, we performed a reformatting and made corrections in the manuscript as shown in Fig. R4. Thank you again for your positive comments and valuable suggestions to improve the quality of our manuscript.

Fig. R4.Analytical image of TENG produced by nylon 11 and PVDF-BTONWs. (a) Physical image of TENG. (b) Surface morphology of nylon-11 fibres .(c) Surface morphology of BTONWs -PVDF. (d)SEM images of BTONWs doped with different mass ratios. (e)Magnified images of BTONWs doped with different mass ratios. (f)XRD image of BTONWs. (g) XRD patterns of PVDF, BTONWs-PVDF film, and BTONWs before mixing with PVDF. (h)FTIR absorption spectra of BTONWs-PVDF nanofibres with different BTONWs mass fractions.

**Comment-5**

The resolution and quality of Figures 4 and 5 must be improved.

**Response:** We sincerely thank the reviewer for careful reading. We entered corrected Figures 4 and 5 in the manuscript.

**Comment-6**

This manuscript requires more experimental tests of the stability and degradation of the performance of the TENG.

**Response:** We sincerely thank the reviewer for comments. We agree with the reviewer that practical environmental factors will affect the performance of TENGs. In this work, similar to what has been usually done in the literature, we have measured the voltage stability of nylon11/PVDF-BTONWs based TENG under lab environment, as shown in Fig. R5, which has constant temperature and humidity, for ~30000 cycles, showing its stability.

Fig. R5. The stability of nylon11/PVDF-BTONWs based TENG

**Comment-7**

This manuscript needs experimental results on the effect of the relative humidity on the output voltage of the TENG.

**Response:**We feel great thanks for your professional review work on our article. According to your nice suggestions,We have done experiments related to the effect of humidity and temperature on TENG. Fig. R6. demonstrates the effect of humidity and temperature on the output performance of TENG. (a) illustrates the variation of TENG output voltage over time under humidity levels of 30%, 50%, 70%, and 90%. It can be observed that as humidity increases, the output voltage gradually decreases. This is attributed to the higher humidity levels causing water molecules in the air to form a conductive water film on the material surface, leading to rapid leakage of surface triboelectric charges and weakening the triboelectric effect *(Small, 2024, 2401846)*. (b) shows the output current under different humidity conditions. Similarly, the current significantly decreases in high-humidity environments, further confirming that increased humidity causes charge leakage and reduces charge transport efficiency *(Small, 2024, 2401846)*. (c) depicts the trend of charge quantity with humidity. It can be observed that higher humidity levels result in a notable reduction in charge accumulation, indicating that high humidity suppresses the charge storage capacity of the TENG, thereby reducing its overall output performance *(Small, 2024, 2401846)*. (d)–(f) illustrate the changes in TENG output voltage, current, and charge quantity at temperatures of 30°C, 60°C, and 90°C. As the temperature increases, the TENG maintains stable output performance within a certain range. Particularly at higher temperatures, the TENG output waveforms exhibit good periodicity and stability. The elevated temperature may enhance the flexibility and surface activity of the materials, thereby improving the contact quality between the materials and partially compensating for the electrical performance losses caused by temperature changes *(Advanced Engineering Materials, 2017, 19(12), 1700275)*. This indicates that TENGs have a certain degree of stability and adaptability under fluctuating temperature conditions.

Fig. R6. Effect of Humidity and Temperature on the Electrical Output of TENGs. (a)-(c)Effect of humidity at 30%, 50%, 70%, and 90% on the voltage, current, and charge of TENGs. (d)-(f)Effect of temperature at 30, 60, and 90 on voltage, current, and charge of TENGs.

**Comment-8**

The authors should include a table with the main parameters, advantages, and limitations of the proposed TENG compared to other types of recent TENGs reported in the literature.

**Response:** We sincerely thank the reviewer for your comments. As shown in Table 1, we have updated the table, which we can see that PVDF-BaTiO_3_ has better and superior output performance compared to the previously reported PVDF-based TENGs and modified in the manuscript.

Table 1 Comparison of different dopant materials in TE layers.

| TE layer | Size(cm^2^) | Voc(V) | Isc(μA) | Pd（W/m^2^） | TENG |
| --- | --- | --- | --- | --- | --- |
| PVDF-none | 2.0*2.0 | 22 | 0.6 | - | [29] |
| PVDF-ZnO | 2.5*3.0 | 119 | 1.6 | 0.11 | [30] |
| PVDF-MXene | 2.0*2.0 | 46 | 2.4 | 0.29 | [31] |
| PVDF-CoFe_2_O_4_ | 2.0*2.0 | 17.2 | 2.27 | 0.09 | [32] |
| PVDF-BaTiO_3_(this work) | 4.0*4.0 | 280 | 12 | 1.45 | - |

**Comment-9**

What are the main challenges of the proposed TENG?

**Response:** We feel great thanks for your professional review work on our article. Overall, there is a great market potential to commercialise TENG, which can be significantly reduced through low-cost material development, process optimisation, environmental adaptation improvements and market positioning adjustments. From a technical point of view, TENG already has a relatively established technology, and the spraying technique has been shown to be more cost-effective in batch production while maintaining similar nanostructured properties *(Advanced Materials Interfaces, 2019, 6(15), 1900557)*. Similarly, the addition of graphene-reinforced composites not only improves wear resistance, but also extends the lifetime to 1 million cycles of operation *(Nano Energy, 2020, 67, 104258)*. In terms of materials, expensive polymers can be replaced with natural materials such as cellulose, chitosan and other bio-based materials. Recent studies have shown that TENGs prepared by using discarded plastic bottles or milk cartons not only have superior performance, but also have extremely low raw material costs *(Environmental Science & Technology, 2021, 55(6), 3712-3721)*. The challenge is mainly in the lack of stability of TENG. As researchers continue to study, TENG is expected to achieve large-scale applications in wearable devices, smart homes and industrial monitoring.

**Comment-10**

What are the future research work?

**Response:**We feel great thanks for your professional review work on our article again. Although the basic material of TENG is completed in the text, using Nylon 11 with PVDF-BTONWs, it is possible to make improvements in the structural aspects and stability of TENG based on recent literature. For example, making a porous structure improves the effective contact surface, and through chemical modification, researchers can introduce functional groups with higher friction electrical sequence positions, which improves the material's charge generation capability. And through surface roughening, the contact area of the material can be increased to further improve the charge transfer efficiency.In 2021, Van-Tien Bui et al. fabricated a bird's nest structure of TENG, and the altered structure doubled the output electrical properties *(Nano Energy,2020, 71, 104561)*. In the same year, Cheedarala used sand to polish the surface of the friction layer, which improved its electrical properties by 40%, but this structure was inferior to the hole structure due to the modification of the surface structure only *(International Journal of Smart and Nano Materials, 2019, 11(1), 9)*. Doldet Tantraviwat listed about the effect of the three types of structures, namely, holes, columns, and flakes, on the PDMS-based TENG, and the experimental results showed that the hole-containing structure has the most optimised electrical output of TENG *(Nano Energy ,2019, 67)*. Secondly, the durability can be increased by spraying compounds, etc. TENGs are prone to failure of the friction electric layer during constant contact separation, which is due to the fact that the friction layer is easy to stick together with the fibres formed by electrostatic spinning, so the TENGs usually decay within a short period of time during the pressing process. Xiang Gang et al. proposed a TENG made of ethyl cellulose wrapped nylon11 and PTFE wrapped PVDF, which can resist 100,000 presses and have significantly enhanced mechanical strength *(ACS Applied Materials And Interfaces, 2023 15.45:52696-52704)*. In addition, Yoonsang Ra was sandblasted with a metallic layer on the friction surface, with an amazing durability of 150,000 presses *(Environ. Technol. Innov. 2021, 24, 102049)*.

**Comment-11**

The conclusions must be enhanced based on the previous comments

**Response:** We thank the reviewer for your comments. We tried our best to improve the conclusions. And we marked in red in the revised paper. In summary, we developed a new method to prepare nanocomposite capacitors with high performance energy harvesting and continuous output. Experimentally, it was demonstrated that BTONWs can improve the electrical output of nanocomposites. The dielectric constant increases by 54.2% at 2%wt doping compared to pure PVDF. To the best of our knowledge, this electrical output is the highest reported in the literature for previously PVDF-based TENGs. In addition, the nanocomposite material illuminates 120 LEDs, enables the calculator and timer to function through a simple external circuit, and illuminates the LEDs simultaneously when the TENG is continuously tapped. The experiments show that the new composite TENG can be used as a micro-generator and the fabric-based generator has good electrical properties.In conclusion, the nylon-11/BTONWs-PVDF micro-nanogenerator has the advantages of being lightweight, portable, self-powered, and inexpensive, which can have a good prospect for application in flexible sensors and wearable devices.

We appreciate for Editors warm work earnestly and hope that the correction will meet with approval.

-------------------------------------------------------------------------------------------------------

**Response to Reviewer 2**

**Comment-1**

Why dielectric constant at higher wt% doping decreases but in general it increases. Explain properly?

**Response:**We feel great thanks for your professional review work on our article. As you are concerned, the interface between the filler and the matrix material may induce significant interfacial polarisation in composites. This polarisation effect may be optimal at a specific filler content (e.g. 2 wt%), resulting in a peak in the dielectric constant. When the filler content is further increased, the interfacial polarisation effect may be masked by the nature of the filler itself, resulting in the dielectric constant not continuing to increase or even decreasing. At low BaTiO₃ content, the BaTiO₃ nanowires may be uniformly distributed in the PVDF matrix, forming a good interface and polarisation. However, as the BaTiO₃ content increases, agglomeration may occur between the nanowires, leading to the disruption of the uniform distribution, which reduces the overall polarisation of the composite, and hence the dielectric constant. At 2 wt%, the material may be in a more optimised state of orientation and polarisation that maximises the dielectric contribution of BaTiO₃. Whereas when the BaTiO₃ content increases, the orientation and polarisation of the material may be difficult to optimise further due to the limitations of the electrostatic spinning process, resulting in a decrease in the dielectric constant.

According to the experimental analysis, the content of BaTiO3 of 2 wt% has reached the optimum output electrical properties, and when the content of BaTiO₃ exceeds a certain percentage, the nanoparticles may agglomerate, resulting in uneven dispersion. This agglomeration phenomenon not only reduces the effective contact area of the material, but may also lead to a decrease in the mechanical and electrical properties of the material, which was observed by TEM at a BaTiO3 content of 3 wt%, and the agglomeration phenomenon becomes more severe when the BaTiO₃ doping reaches 4 wt%, which undoubtedly leads to an extreme decrease in the output electrical properties*(Prasad G, Adonijah S，2023, Nano Energy, 108, 108178)*. In short, Excessive doping can lead to degradation of properties, such as fluctuations in

lectrical properties, phase separation phenomena and degradation of mechanical properties. For this purpose, we carried out doping experiments with 4% BTONWs, and the results are shown in the Fig. R7. In fact, we have several sets of experiments in the experimental process, according to the figure shows that 4wt% BaTiO3 is lower than 3wt% in the electrical output, from the data also indicates that the content is increasing in the process of the performance is decreasing.

Fig. R7. Output electrical properties of 4wt% BaTiO3 doping

**Comment-2**

In XRD after 20 there is additional peak which is not index must be given reason for occurrence. The lattice parameters must be given properly? Charging of capacitor and discharge for Figure 5 g, h, i can be added?

**Response:** We think this is an excellent comment. In the XRD pattern, the additional peak appearing after 20° was not indexed by conventional diffraction peaks as shown in Fig. R8(a). According to the results of the JADE analysis software, this peak maybe caused by the intermediate product Ba₄Ti₂O_27_. This intermediate product may be generated during synthesis at high temperature under certain reaction conditions and coexist with BaTiO₃.The crystal structure of Ba₄Ti₂O_27_ is a tetragonal crystal system (space group: C2/m) with the lattice parameters: a = 1.9823, b = 1.1394, c = 0.991 *( Nanoscale, 2018, 10(37): 17751-60)*.

In response to your reference to the capacitor charging and discharging parts of Fig. R8. (c,d,e), the experiments in these figures actually do not drive the LEDs by capacitor charging and discharging, but by converting them to DC via a full-bridge rectifier. Fig. R8(c) Timer capacitor charging and discharging as shown in Fig. R8(b). This charge/discharge curve not only demonstrates the basic working principle of capacitor and TENG, but also provides important experimental data and design basis for optimizing the energy harvesting and power supply system.

Fig. R8. (a) FTIR absorption spectra of BTONWs-PVDF nanofibres with different BTONWs mass fractions. (b)Timer capacitor charging and discharging. (c) a timer in working condition. (d) A yellow LED and (e) a green LED to be lighted.

**Comment-3**

Working mechanism has some error the arrow direction for flow of current is same., Also added the arrow for flow of electrons as well.

**Response:** We sincerely thank the reviewer for careful reading. Fig. R9(a) shows a schematic diagram of the device and the fabricated TENG. When the nylon-11 and the PVDF-BTONWs are contacted under pressure, a surface charge transfer occurs in the contact area due to the triboelectric effect, where the positively charged layer of the nylon-11 (top) loses electrons to carry a positive charge, while the negatively charged layer of the PVDF-BTONWs (bottom) gains electrons to carry a negative charge. After the separation of the nylon-11 and the PVDF-BTONWs, a potential difference between the top and bottom electrodes occurs due to electrostatic induction, which induces electrons to flow from the bottom electrode to the top electrode, forming a half-cycle negative current pulse. When the nylon-11 and PVDF-BTONWs are pressed, a reverse current pulse, i.e., a half-cycle positive current pulse, is generated. Repeatedly， the external circuit could generate AC during each pressing and releasing cycle, as shown in Fig. R9(b). The manuscript has been revised accordingly with red.

Fig. R9. (a) Schematic diagram of the device assembly structure. (b) The working mechanism of TENG.

**Comment-4**

Electrical output of TENG must be optimize at various air gap. How mechanical properties of the composite effect the performance of TENG.

**Response:** We sincerely thank the reviewer for comments. Following the reviewer’s suggestion, the output performance of the TENG is characterized under different device size, frequency, and separation distance, as shown in Fig. R10(a-i). With the increase of the device size, the peak values of V_OC_, I_SC_, and Q_C_ exhibit an increasing trend accordingly, as shown in Fig. R10(a-c). This phenomenon is ascribed to the enhanced capacitance of the TENG with increasing the device size. The influence of the frequency on the performance of the TENG is also studied. The output performance including V_OC_, I_SC_, and Q_C_ are enhanced with increasing the frequency. This is primarily due to the reduction in the duration of the current peak at higher frequency, which increases the transfer rate of friction charges and the amplitude of I_SC_ and Q_C_. Accordingly, external electrons flow within a shorter duration to reach equilibrium, thereby enhancing the V_OC_. Moreover, the separation distance is another key factor influencing the output performance of TENG. The V_OC_, I_SC_, and Q_C_ increase with increasing the separation distance. Particularly, the output characteristics exhibit linear relationship with the separation distance, which is ascribed to the enhanced triboelectrification effect with increasing separation distance. Therefore, these results indicate that the output performance of the TENG is closely associated with the device size, frequency, and air gap.

Fig. R10. V_OC_, I_SC_, and Q_C_ of the TENG with an increase of (a-c) device size, (d-f) frequency, and (g-i) separation distance.

**Comment-5**

Why PVDF was chosen inspite of PVDF-TrFE as beta phase is proven to be higher in PVDF-TrFE. How the authors optimize this electric field for there electrospinning 21 KV?

**Response:** We think this is an excellent suggestion. Considering that PVDF-TrFE has a higher *β*-phase content, its manufacturing process may be more complex and costly.PVDF may be more cost-effective as a more economical material, especially in large-scale production and applications. Therefore, despite the superior performance of PVDF-TrFE, PVDF was chosen for its lower cost and wide availability.In addition, by optimizing the electrostatic spinning process, we were able to achieve a higher percentage of *β*-phase crystals in PVDF fibers, thus bridging the performance gap between them and PVDF-TrFE to some extent.

Fig. R11. SEM of PVDF at 10KV,15KV voltage

As shown in Fig. R11, the voltage during the electrostatic spinning process has a significant effect on the fiber quality. At lower voltages (e.g., 10 KV), bead-like defects tend to appear in the fiber, resulting in unstable spinning quality. With the gradual increase of voltage, the bead defects gradually decrease and the fiber surface gradually becomes smooth. When the voltage reaches 15 KV, the spun fiber has been more uniform, but still has not reached the best state. In the experiment, we finally determined 21 KV as the optimal electrostatic spinning voltage by adjusting the laboratory parameters (including humidity, temperature, and spinning distance) and combining them with the optimization results during the spinning process. Under this condition, the fibers are not only uniform and smooth, but also have high mechanical strength and good piezoelectric properties.

**Comment-6**

Important references in TENG need to be added such as: Nano Energy 131, 110319, 2024; Journal of Materiomics 10 (4), 792-802, 2024; Journal of Science: Advanced Materials and Devices 9 (2), 100693, 2024 and Nano Energy 101, 107620, 2022.

**Response:** We sincerely appreciate the valuable comments. We have checked the literature carefully and added more references in the revised manuscript.

**Comment-7**

English grammertical errors need to be removed

**Response:** We were really sorry for our careless mistakes. Thank you for your reminder.

We sincerely thank the reviewer for careful reading. As suggested by the reviewer. We update the manuscript.

-------------------------------------------------------------------------------------------------------

**Response to Reviewer 3**

**Comment-1**

Could the authors provide quantitative comparisons with existing TENG systems to highlight the novelty of achieving a power -density of 1.45 W/m²?

**Response:** We sincerely thank the reviewer for careful reading. As shown in Table Ⅰ, we have updated the table to include references, from which we can see that PVDF-BaTiO_3_ has better and superior output performance compared to the previously reported PVDF-based TENGs .Although the size of each TENG is different, it can be seen that the efficiency of the TENG produced by us is much higher than that produced by other PVDF TENG.

Table Ⅰ. Comparison of different dopant materials in TE layers.

| TE layer | Size(cm^2^) | Voc(V) | Isc(μA) | Pd（W/m^2^） | TENG |
| --- | --- | --- | --- | --- | --- |
| PVDF-none | 2.0*2.0 | 22 | 0.6 | - | [29] |
| PVDF-ZnO | 2.5*3.0 | 119 | 1.6 | 0.11 | [30] |
| PVDF-MXene | 2.0*2.0 | 46 | 2.4 | 0.29 | [31] |
| PVDF-CoFe_2_O_4_ | 2.0*2.0 | 17.2 | 2.27 | 0.09 | [32] |
| PVDF-BaTiO_3_(this work) | 4.0*4.0 | 280 | 12 | 1.45 | - |

**Comment-2**

Why was 2 wt% BaTiO₃ selected as the optimal doping ratio, and how does it compare with other ratios in terms of performance trade-offs?

**Response:** We sincerely appreciate the valuable comments. The performance metrics of a TENG with varying weight percentages of a material, likely BTONWs mixed with PVDF as revealed in Fig. R12. The BaTiO₃ agglomeration results in deterioration of contacts between the triboelectric layers and degradation of output performance, akin to the blocking effect of BN and Fe_3_O_4_ additives on PVDF. (*ACS Applied Materials & Interfaces 2018, 10, 25660; ACS Applied Nano Materials 2022, 5, 16906*).

Fig. R12. BTONWs-PVDF electrical output image. Image of (a) Short-circuit current. (b) Open-circuit voltage. (c) Transfer charge quantity. (d)Dielectric constant.

**Comment-3**

Could the authors elaborate on the challenges associated with scaling TENG technology for commercial applications, especially in terms of material costs and production feasibility?

**Response:** We thank the reviewer for professional comments. Overall, there is a great market potential to commercialise TENG, which can be significantly reduced through low-cost material development, process optimisation, environmental adaptation improvements and market positioning adjustments. From a technical point of view, TENG already has a relatively established technology, and the spraying technique has been shown to be more cost-effective in batch production while maintaining similar nanostructured properties (*Advanced Materials Interfaces, 2019, 6(15), 1900557*). Similarly, the addition of graphene-reinforced composites not only improves wear resistance, but also extends the lifetime to 1 million cycles of operation (*Nano Energy, 2020, 67, 104258*). In terms of materials, expensive polymers can be replaced with natural materials such as cellulose, chitosan and other bio-based materials. Recent studies have shown that TENGs prepared by using discarded plastic bottles or milk cartons not only have superior performance, but also have extremely low raw material costs (*Environmental Science & Technology, 2021,55(6), 3712-3721*). The challenge is mainly in the lack of stability of TENG. As researchers continue to study, TENG is expected to achieve large-scale applications in wearable devices, smart homes and industrial monitoring.

**Comment-4**

How does the authors findings address existing gaps in TENG performance and durability for wearable and lowpower electronic applications?

**Response:** We feel great thanks for your professional review work on our article. The weaknesses of wearable devices and low power applications restrict their development and practicality, with limited output performance, insufficient flexibility and comfort, and insufficient mechanical durability. TENGs typically have low output current densities and power densities, making it difficult to meet the demands of high-energy wearable devices. The friction layer materials have insufficient charge separation capability and low charge injection and transfer efficiency.

Nylon-11 is often used as a friction layer due to elasticity and mechanical stability, while the PVDF-BaTiO₃ composites have excellent polarization characteristics.The PVDF-BaTiO₃ enhances the charge separation capability, which significantly increases the output voltage and current density of the TENG. The high frictional charge density property of nylon-11 further optimizes the performance of the friction layer (*Nano Energy, 2023, 85, 105947*). Second, wearable devices require high material flexibility, but some rigid materials (e.g., inorganic fillers) used in existing TENGs reduce the flexibility of the devices (*Nano Research, 2023, 13(8), 2038-2045*). Ergonomic needs are not adequately considered in the design of friction layers and electrode materials. It affects the wearing experience and may lead to user discomfort. Nylon-11 has excellent mechanical flexibility to maintain stable in bending situations.The composite structure of PVDF-BaTiO₃ provides additional mechanical durability and avoids the problem of fatigue cracking during prolonged use (*Nature Reviews Materials, 2023, 4(5), 377-389*). The wear-resistant properties of nylon-11 reduced the surface wear rate.The inorganic particles in the PVDF-BaTiO₃ composites enhanced the aging resistance of the overall material (*Nano-Micro Letters, 2023, 14(1), 10*). Nylon-11 and PVDF-BaTiO₃ TENGs are expected to solve the defects in the existing wearable devices.

**Comment-5**

Could the authors include the environmental conditions (e.g., temperature, humidity) under which TENG performance was tested to ensure reproducibility?

**Response:** We sincerely thank the reviewer for comments. Fig. R13. demonstrates the effect of humidity and temperature on the output performance of TENG. (a) illustrates the variation of TENG output voltage over time under humidity levels of 30%, 50%, 70%, and 90%. It can be observed that as humidity increases, the output voltage gradually decreases. This is attributed to the higher humidity levels causing water molecules in the air to form a conductive water film on the material surface, leading to rapid leakage of surface triboelectric charges and weakening the triboelectric effect *(Small, 2024, 2401846)*. (b) shows the output current under different humidity conditions. Similarly, the current significantly decreases in high-humidity environments, further confirming that increased humidity causes charge leakage and reduces charge transport efficiency *(Small, 2024, 2401846)*. (c) depicts the trend of charge quantity with humidity. It can be observed that higher humidity levels result in a notable reduction in charge accumulation, indicating that high humidity suppresses the charge storage capacity of the TENG, thereby reducing its overall output performance *(Small, 2024, 2401846)*. (d)–(f) illustrate the changes in TENG output voltage, current, and charge quantity at temperatures of 30°C, 60°C, and 90°C. As the temperature increases, the TENG maintains stable output performance within a certain range. Particularly at higher temperatures, the TENG output waveforms exhibit good periodicity and stability. The elevated temperature may enhance the flexibility and surface activity of the materials, thereby improving the contact quality between the materials and partially compensating for the electrical performance losses caused by temperature changes *(Advanced Engineering Materials, 2017, 19(12), 1700275)*. This indicates that TENGs have a certain degree of stability and adaptability under fluctuating temperature conditions.

Fig. R13. Effect of Humidity and Temperature on the Electrical Output of TENGs. (a)-(c)Effect of humidity at 30%, 50%, 70%, and 90% on the voltage, current, and charge of TENGs. (d)-(f)Effect of temperature at 30, 60, and 90 on voltage, current, and charge of TENGs.

**Comment-6**

Could the authors explain the rationale for excluding BaTiO₃ doping ratios above 3 wt%, and were any preliminary tests conducted at higher ratios?

**Response:** We sincerely thank the editor and all reviewers for their valuable feedback that we have used to improve the quality of our manuscript. In composites, the interface between the filler and the matrix material may induce significant interfacial polarisation. This polarisation effect may be optimal at a specific filler content (e.g. 2 wt%), resulting in a peak in the dielectric constant. When the filler content is further increased, the interfacial polarisation effect may be masked by the nature of the filler itself, resulting in the dielectric constant not continuing to increase or even decreasing. At low BaTiO_3_ content, the BaTiO_3_ nanowires may be uniformly distributed in the PVDF matrix, forming a good interface and polarisation. However, as the BaTiO_3_ content increases, agglomeration may occur between the nanowires, leading to the disruption of the uniform distribution, which reduces the overall polarisation of the composite, and hence the dielectric constant. At 2 wt%, the material may be in a more optimised state of orientation and polarisation that maximises the dielectric contribution of BaTiO_3_. Whereas when the BaTiO_3_ content increases, the orientation and polarisation of the material may be difficult to optimise further due to the limitations of the electrostatic spinning process, resulting in a decrease in the dielectric constant.

According to the experimental analysis, the content of BaTiO_3_ of 2wt% has reached the optimum output electrical properties, and when the content of BaTiO₃ exceeds a certain percentage, the nanoparticles may agglomerate, resulting in uneven dispersion. This agglomeration phenomenon not only reduces the effective contact area of the material, but may also lead to a decrease in the mechanical and electrical properties of the material, which was observed by TEM at a BaTiO_3_ content of 3 wt%, and the agglomeration phenomenon becomes more severe when the BaTiO_3_ doping reaches 4 wt%, which undoubtedly leads to an extreme decrease in the output electrical properties(*Nano Energy, 2023, 108, 108178*). In short, Excessive doping can lead to degradation of properties, such as fluctuations in electrical properties, phase separation phenomena and degradation of mechanical properties. For this purpose, we carried out doping experiments with 4% BTONWs, and the results are shown in the Fig. R14. In fact, we have several sets of experiments in the experimental process, according to the figure shows that 4wt% BaTiO_3_ is lower than 3wt% in the electrical output, from the data also indicates that the content is increasing in the process of the performance is decreasing.

Fig. R14. Output electrical properties of 4wt% BaTiO3 doping

**Comment-7**

Can the authors provide more details about the electrospinning parameters, such as the material of the syringe and adjustments made to flow rates and drum speed during the fabrication process?

**Response:** We appreciate the thoroughness of the review's thinking, and we have listed all the details. Pour the precursor into a 10 mL syringe made with a polypropylene barrel, 20 G stainless steel needle, and PTFE tubing. Injections were made at a rate of 1 ml per hour on a syringe pump. The needle was mounted on a reciprocating moving platform and a voltage of 21 KV was applied to the needle with a distance of 17 cm from the needle to the drum collector for collection, and the drum collector was rotated at 1500 rpm. The syringe pump was a dual-channel micro syringe pump WZS-50F6 made by Zhejiang Smith Medical Instrument Co. Ltd, and the reciprocating platform and drum collector were made by Qingdao Nuokang Environmental Protection Technology Co.

**Comment-8**

How does the environmental sustainability of your materials and processes compare to other TENG fabrication methods?

**Response:** We appreciate the thoroughness of the review's thinking. Compared to other TENG fabrication methods, our materials and processes demonstrate significant advantages in terms of environmental sustainability. First, we have selected environmentally friendly materials such as PVDF and Nylon-11, which not only exhibit excellent electrical properties but also minimize potential environmental impacts*(Journal of Applied Physics, 2022, 131(16))*. Second, we have minimized the use of toxic reagents during the fabrication process and optimized the workflow to reduce energy consumption and waste generation. Furthermore, compared to traditional TENG fabrication methods, our approach places greater emphasis on the recyclability and reusability of materials, thereby further mitigating adverse environmental effects. This design philosophy, which balances high performance with environmental protection, enables our TENG to achieve efficient energy output while contributing to the goals of sustainable development.

**Comment-9**

Could the authors improve the clarity and resolution of Figures 3 and 5 to make the structural details and annotations more legible?

**Response:** We sincerely thank the reviewer for careful reading. We entered corrected Figures 3 and 5 in the manuscript.

**Comment-10**

Would the authors consider adding a comparative table or schematic summarizing the electrical performance of TENGs with different material compositions for easier reference?

**Response:** We genuinely thank the reviewer for comments. Please refer to the response to Comment-1.

**Comment-11**

Could the authors expand on the interaction between the dielectric constant, mechanical stability, and electrical output at different BaTiO₃ doping ratios, especially above 2 wt%?

**Response:** We thank the reviewer for professional comments. The dielectric constant is a critical indicator of a material's ability to respond to an external electric field, and a higher dielectric constant generally enhances the energy storage capacity of the material. However, when the doping content of BaTiO₃ exceeds 2 wt%, although the dielectric constant increases, its enhancement of electrical performance is not linear. This may be attributed to an excessively high dielectric constant causing the shielding effect of interfacial charges, which suppresses the separation and transfer efficiency of triboelectric charges *(Nano Energy, 2024, 123: 109383)*. In TENG devices, the mechanical stability of materials is crucial for triboelectric output. When the BaTiO₃ doping content exceeds 2 wt%, BaTiO₃ nanowires may aggregate within the matrix, leading to a decrease in material uniformity and degradation of mechanical properties *(Frontiers of Materials Science, 2021, 15(1): 79–97)*. The deterioration of mechanical properties reduces the quality of contact during frictional processes, thereby indirectly affecting output performance. At higher BaTiO₃ doping levels, although the dielectric constant increases, the decline in mechanical properties and the interfacial charge shielding effect may result in reduced electrical output performance *(Nano Energy, 2019, 62: 620–627)*. This indicates that at excessively high doping levels, electrical output and dielectric constant are not simply positively correlated. Below 2 wt%, BaTiO₃ nanowires exhibit more uniform distribution, and interfacial polarization effects are more pronounced, effectively enhancing dielectric performance and triboelectric output. In contrast, above 2 wt%, nanowire aggregation weakens the contribution of interfacial polarization while increasing the risk of mechanical instability.

**Comment-12**

Have the authors tested the long-term stability and degradation of the TENG under continuous operation, and if so, could you discuss the results?

**Response:** We thank the reviewer for comprehensive thinking. We agree with the reviewer that practical environmental factors will affect the performance of TENGs. In this work, similar to what has been usually done in the literature, we have measured the voltage stability of nylon11/PVDF-BTONWs based TENG under lab environment, as shown in Fig. R15, which has constant temperature and humidity, for ~30000 cycles, showing its stability.

Fig. R15. The stability of nylon11/PVDF-BTONWs based TENG

**Comment-13**

Can the authors provide further insights into the scalability of your approach for large-scale production, including potential challenges or cost analyses?

**Response:** We sincerely thank the reviewer for recognizing our products. The large-scale production of nylon-11 /PVDF-BTONWs TENGs show significant potential for scalability, but it still faces several key challenges and cost-related issues. First, the composite materials of nylon-11/PVDF-BTONWs offer remarkable performance improvements for TENG output, but their synthesis processes are complex, involving the preparation of functionalized nanomaterials (such as BTONWs), which are costly and require sophisticated equipment *(Advanced Materials,2018, 30(19), 1706790).* Moreover, the availability and processing stability of these materials remain major constraints for large-scale production, as high-quality PVDF-BTONWs typically rely on precisely controlled chemical processes, which are difficult to standardize and scale in industrial settings *(Nano Energy, 2014, 10, 513–519).* In addition, the fabrication of TENGs to meet the needs of flexible applications, such as wearable devices, often involves complex micro-nano-structuring processes, such as electrospinning or nano-patterning, further increasing manufacturing costs and technical challenges (*Advanced Materials,2020, 32(22), 2001663*). While simplified methods, such as spraying, printing, and lamination, could reduce some costs, ensuring consistent performance and yield during production remains an unresolved issue (*Nano Research, 2020, 13(8), 2038–2045*). Furthermore, life cycle assessments suggest that TENG production may involve the use of non-degradable waste or solvents, thereby raising environmental concerns (*Nano-Micro Letters, 2020, 14(1), 15*). To achieve economic feasibility in large-scale production, further development of low-cost and sustainable materials, as well as optimization of manufacturing processes to meet industrial standards, is needed, alongside a comprehensive life cycle analysis to promote green production practices (*Advanced Materials, 2021, 33(50), 2100833).* TENGs need further development in large-scale production and may be realized in the future.

**Comment-14**

How do the authors envision the practical implementation of your TENG for powering wearable devices or lowpower electronics, given the current state of the technology?

**Response:** We thank the reviewer for comments. Given the current technological status, the practical implementation of nylon-11/PVDF-BTONWs TENGs for powering wearable or low-power electronic devices involves several considerations and solutions to existing challenges. First, the composite materials of nylon-11 and PVDF-BTONWs show significant potential for energy harvesting, especially in environments with frequent mechanical motion, such as wearable applications. These materials offer good mechanical flexibility, high triboelectric output, and stable performance, all of which are crucial for the successful integration of TENGs into wearable devices (*Advanced Energy Materials, 2021, 11(8), 2002744*). For energy harvesting, wearable devices could use TENGs as a micro-power source, collecting energy from mechanical movements such as walking, bending, or even breathing. The high flexibility of nylon-11 combined with the enhanced triboelectric effect of PVDF-BTONWs enables efficient energy generation from small-scale, low-frequency motions. For example, wearable bands or patches with embedded TENGs could harvest energy from arm movements and store it in supercapacitors or small batteries *(Advanced Materials, 2020, 32(19), 1908485*). The integration of PVDF-BTONWs further improves energy conversion efficiency, particularly in terms of mechanical deformation and surface charge generation *(Journal of Materials Chemistry A, 2023 11(6), 3133-3142).*

A key factor for practical implementation is optimizing TENG design to ensure sufficient power output for low-power electronics, such as sensors, LED lights, or small wireless devices. One possible approach is to integrate multiple TENG units in parallel or series within wearable devices to enhance overall power output, which is essential for devices that require continuous or reliable operation *(Nature Communications, 2021, 12(1),5177)*. Additionally, power management circuits are needed to regulate the energy harvested by the TENG and effectively store it in energy storage systems, such as capacitors or batteries, ensuring stable power supply *(Nano-Micro Letters, 2020, 14(1), 1-15).* However, the implementation of nylon-11/PVDF-BTONWs TENGs in wearable devices faces several challenges. These include scalability in material production, long-term stability and durability of the devices, and reducing manufacturing costs *(Nano Energy, 2022， 90, 106497)*. The mechanical robustness of TENGs must also be tested over long periods of use to ensure they do not significantly degrade with repeated deformation. Furthermore, the power density of TENGs needs to be optimized to meet the energy demands of modern wearable electronic devices, which are typically low-power but require continuous or on-demand energy harvesting *(Nano Energy, 2021， 80, 105515)*.

**Comment-15**

Would the authors consider correcting typographical errors (e.g., "couldnot" to "could not" and “probles” in the 4th paragraph in the introduction section) and ensuring consistent use of technical terms throughout the manuscript?

**Response:** We were really sorry for our careless mistakes. Thank you for your reminder. We sincerely thank the reviewer for careful reading. As suggested by the reviewer. We update the manuscript.

**Comment-16**

Could the authors ensure the figure captions align with the journal’s formatting guidelines for clarity and consistency?

**Response:** We thank the reviewer for comments. We have changed all figures, tables, etc. in the manuscript according to the journal’s formatting guidelines.

**Comment-17**

Could the authors clarify the abbreviation “PDMS and PMMA” in the beginning of the manuscript.

**Response:** We sincerely thank the reviewers for their careful review of the manuscript. We have revised the manuscript by adding the full names PDMS and PMMA at the beginning of the manuscript with red.

**Comment-18**

Are all your references up-to-date, particularly regarding recent advancements in triboelectric nanogenerators and their applications?

**Response:** We thank the reviewer for comments.The references cited in this paper contain the most recent literature from recent years. In particular, more previous classical literature on material properties is used as support. The inspiration for this paper comes from the literature on the application of TENG in the last 5 years.

**Comment-19**

Could you include more references on the scalability and commercialization of TENGs to provide additional context for your study?

**Response:** We thank the reviewer for comprehensive thinking. We have updated the references in the manuscript about the scalability and commercialization of TENGs.

We appreciate for Editors warm work earnestly and hope that the correction will meet with approval.
